# Taurine as a potential anti-ageing therapy: the key to reversing the ageing process? Short communication

**DOI:** 10.1097/MS9.0000000000000826

**Published:** 2023-05-20

**Authors:** Ayesha Sheikh, Maham Iqbal

**Affiliations:** Department of Medicine, Dow University of Health Sciences, Karachi, Sindh, Pakistan

**Keywords:** ageing, free radicals, reactive oxygen species, taurine

## Abstract

Taurine supplementation may be a viable solution to the problem of our cells manufacturing potentially hazardous by-products known as ‘free radicals’. Some of these chemicals serve crucial biological activities, but excessive amounts can harm internal cell structures, reducing the cells’ capacity to operate. The regulatory systems that contribute to maintaining a healthy balance of reactive oxygen species in the body deteriorate with age. Thus, in this article, we examine how the amino acid taurine could be used in anti-ageing therapy, as well as its mechanism of action, consequences and suggestions.

Ageing is defined as a process that decreases the human body’s capacity to withstand damage, disease and stress. It affects physiological processes on many levels, including respiratory cycles, vision, blood pressure, postural dynamics and ultimately raises the risk of mortality and lowers fertility^1,2^. Recent studies have revealed that ageing is caused by the build-up of cellular and molecular damage^3^. This damage is mostly caused by reactions involving free radicals and other reactive oxygen species, as well as reactions involving sugars and reactive aldehydes and spontaneous mistakes in metabolic processes^4^. Oxygen metabolism and adenosine triphosphate (ATP) synthesis primarily produce reactive oxygen species (ROS) as a by-product^1^. Excessive ROS are produced as a result of mitochondrial failure and antioxidant system degeneration in ageing, which causes cell dysfunction and death, for example cell cycle arrest, apoptosis and necrosis^5^. Along with metabolic alterations, abnormal oncogene activities, DNA damage accumulation and telomere shortening, ROS also causes the multiplication of senescent cells as a stress response in ageing^1^. These deficits play a role in the development of cancer, cardiovascular disease, metabolic disorders, exercise intolerance and neurological disorders, which have led to an increased incidence of psychological disorders, including depression and anxiety, along with a gradually decreasing quality of life in the elderly population^5,6^.

Dietary interventions such as caloric restriction, intermittent fasting and time-restricted eating might slow down or reverse ageing. Pharmacological interventions include telomerase to prevent telomere shortening, probiotics that target the microbiota and have been shown to effectively promote microbial community balance in the body in older adults and induce numerous health advantages, and medications that mimic the advantages of calorie restriction on long life without requiring significant changes in lifestyle^7^. Antioxidant dietary supplementation is also growing in popularity as an anti-ageing therapy. However, their molecular pathways for oxidative stress defence and anti-ageing actions are not fully understood^4^.

Taurine 2-aminoethanesulphonic acid, a non-essential amino acid containing sulphur, is widely expressed in animal tissues such as the brain, muscles, retina and organs throughout the body^7^. It has a special sulphonic acid constitution that affects cellular processes like osmotic adjustment, anti-oxidation, ion transport regulation and bile acid conjugation^8^. The use of dietary antioxidants has been studied as one method to enhance the body’s capacity to combat oxidative stress. Due to its capacity to neutralise hypochlorous acid, a highly hazardous oxidant produced by neutrophils in systemic inflammation in humans, it has been utilised as an efficient antioxidant^9^. Furthermore, hypotaurine, a precursor of taurine, can scavenge hydroxyl radicals and block the autoxidation of iron ions, minimising peroxidation events of lipids^10^. Moreover, as demonstrated in prior studies, the antioxidant activity of taurine can significantly aid in the preservation and stability of mitochondrial functions. Taurine supplementation can significantly reduce mitochondrial dysfunction, which is a significant contributor to age-related illness, as per Jong *et al*.’s study^11^. Its ability to reduce the generation of ROS and boost the activity of antioxidant enzymes like superoxide dismutase (SOD) was noticed. Inhibiting the increased synthesis of proinflammatory oxidants such as superoxide anion is another way that it takes part in the antioxidant process^12^. As a chemical chaperone, it promotes proper protein folding and membrane trafficking of the mutant cystic fibrosis transmembrane conductance regulator (CFTR). Its deficiency in skeletal muscle has been shown to increase ER (endoplasmic reticulum) stress and affect protein homeostasis by activating genes involved in protein synthesis, metabolism and folding. This can result in the accumulation of damaged protein, which can cause cellular ageing^13^.

Even though taurine is a potent antioxidant, there are not many studies that demonstrate how it can delay ageing, particularly in humans. The amino acid taurine may be a useful dietary supplement to delay the onset of ageing, according to a recent study published in the journal *Nutrition* ^9^. Brazilian researchers conducted a double-blind, randomised control trial with 24 female volunteers aged 55–70. They were divided into two groups at random. For 16 weeks, one group took three 500 mg taurine pills daily (1.5 g per day). As a placebo, the other group was given pills that only contained cornstarch^9^. One of the most interesting findings was an almost 20% rise in the antioxidant enzyme SOD levels in the taurine group compared to a 3.5% decline in the control group, implying that taurine could be used to regulate oxidative stress during the process of ageing^9,14^. The outcomes were satisfactory, but researchers believe that a larger taurine dosage, more participants and a longer trial period could offer stronger evidence for its advantages.

Taurine may strengthen your body’s antioxidant defences and reduce your risk of diabetes, high blood pressure and cardiovascular disease^9^. It is naturally created in a few body tissues, particularly the liver, and plays an essential role in the integrity of the central nervous system, immunity, vision and fertility. The inclusion of taurine in the diet may provide a widely available, inexpensive, low-risk method of preventing ageing with results that are better compared to currently available, expensive anti-ageing therapies. Red meat, organ meats, chicken, turkey, eggs, shellfish like scallops, mussels and clams, as well as modern products like fizzy drinks, all contain significant concentrations of taurine^8^. Taurine supplements cannot work wonders on their own; they can only help when additional efforts are made, such as leading a healthy lifestyle with a balanced diet and regular exercise. In conclusion, more long-term prospective cohort studies and randomised controlled trials on a variety of age groups with longer follow-up periods, larger sample sizes, and higher dosages are needed before an unambiguous association with the advantages and disadvantages of taurine supplementation as a dietary antioxidant agent in ageing can be established.

## Ethical approval

This paper did not involve patients; therefore, no ethical approval was required.

## Consent

This study was not done on patients or volunteers; therefore, no written consent was required.

## Sources of funding

No funding was acquired.

## Author contribution

A.S.: conception of the study, drafting of the work, final approval and agree to the accuracy of the work; M.I.: drafting of the work, final approval and agree to the accuracy of the work.

## Conflicts of interest disclosure

The authors declare that there are no conflicts of interest.

## Research registration unique identifying number (UIN)

1. Name of the registry: not applicable.
2. Unique identifying number or registration ID: not applicable.
3. Hyperlink to your specific registration (must be publicly accessible and will be checked): not applicable.

## Guarantor

Ayesha Sheikh and Maham Iqbal.

## Data availability statement

Not applicable.

## Provenance and peer review

Not commissioned, externally peer-reviewed.
